# Effect of CYP3A4*22, CYP3A5*3, and *CYP3A* combined genotypes on tamoxifen metabolism

**DOI:** 10.1007/s00228-017-2323-2

**Published:** 2017-08-28

**Authors:** A. B. Sanchez Spitman, D. J. A. R. Moes, H. Gelderblom, V. O. Dezentje, J.J. Swen, H. J. Guchelaar

**Affiliations:** 1 Leiden Network for Personalised Therapeutics, Department of Clinical Pharmacy and Toxicology, Leiden University Medical Center, Albinusdreef 2, Leiden, 2300 RC The Netherlands; 2 Department of Medical Oncology, Leiden University Medical Center, Leiden, The Netherlands; 3 Department of Medical Oncology, Reinier de Graaf, Delft, The Netherlands

**Keywords:** Tamoxifen, Endoxifen, CYP3A4*22 and CYP3A5*3

## Abstract

**Background:**

Tamoxifen is one of the cornerstones of endocrine therapy for breast cancer. Recently, the decreased activity CYP3A4*22 allele and the loss of function CYP3A5*3 allele have been described as potential factors that could help to explain the inter-patient variability in tamoxifen metabolism. The aim of this study is to investigate the effect of CYP3A4*22, CYP3A5*3, and *CYP3A* combined genotypes on tamoxifen metabolism.

**Methods:**

DNA from 667 women enrolled in the CYPTAM study (NTR1509) was genotyped (*CYP2D6*, CYP3A4*22, and CYP3A5*3). Tamoxifen and metabolite concentrations were measured in serum, and metabolic ratios were calculated. The effect of the CYP3A4*22, CYP3A5*3, and *CYP3A* combined genotypes in addition to the CYP2D6 genotypes was examined by multiple linear regression analysis.

**Results:**

CYP3A4*22 carriers reached significant higher concentrations of tamoxifen, *N*-desmethyl-tamoxifen, and 4-hydroxy-tamoxifen compared to non-carriers, whereas a tendency toward increased endoxifen levels was observed (*p* = 0.088). The metabolic ratio tamoxifen/*N*-desmethyl-tamoxifen was significantly higher in CYP3A4*22 individuals (0.59 vs. 0.52, *p* < 0.001). At the same time, CYP3A4*22 genotype contributed to improving the inter-variability [*R* ^2^ of the (log-transformed) metabolic ratio tamoxifen/*N*-desmethyl-tamoxifen improved from 21.8 to 23.9%, *p* < 0.001]. CYP3A5*3 marginally improved the explained variability of the (log transformed) metabolic ratio 4-hydroxy-tamoxifen/endoxifen (from 44.9 to 46.2%, *p* < 0.038).

**Conclusion:**

Our data demonstrate that CYP3A genotype has a minor effect to explaining the variability between patients in tamoxifen metabolism and has no added value in addition to CYP2D6 genotype.

**Electronic supplementary material:**

The online version of this article (10.1007/s00228-017-2323-2) contains supplementary material, which is available to authorized users.

## Introduction

Breast cancer is the most common diagnosed cancer in women, representing nearly 25% of all cancers [1]. Approximately 60–75% of breast cancer patients have estrogen receptor-positive tumors [2], and in such cases, endocrine therapy may be indicated.

**Fig. 1:**
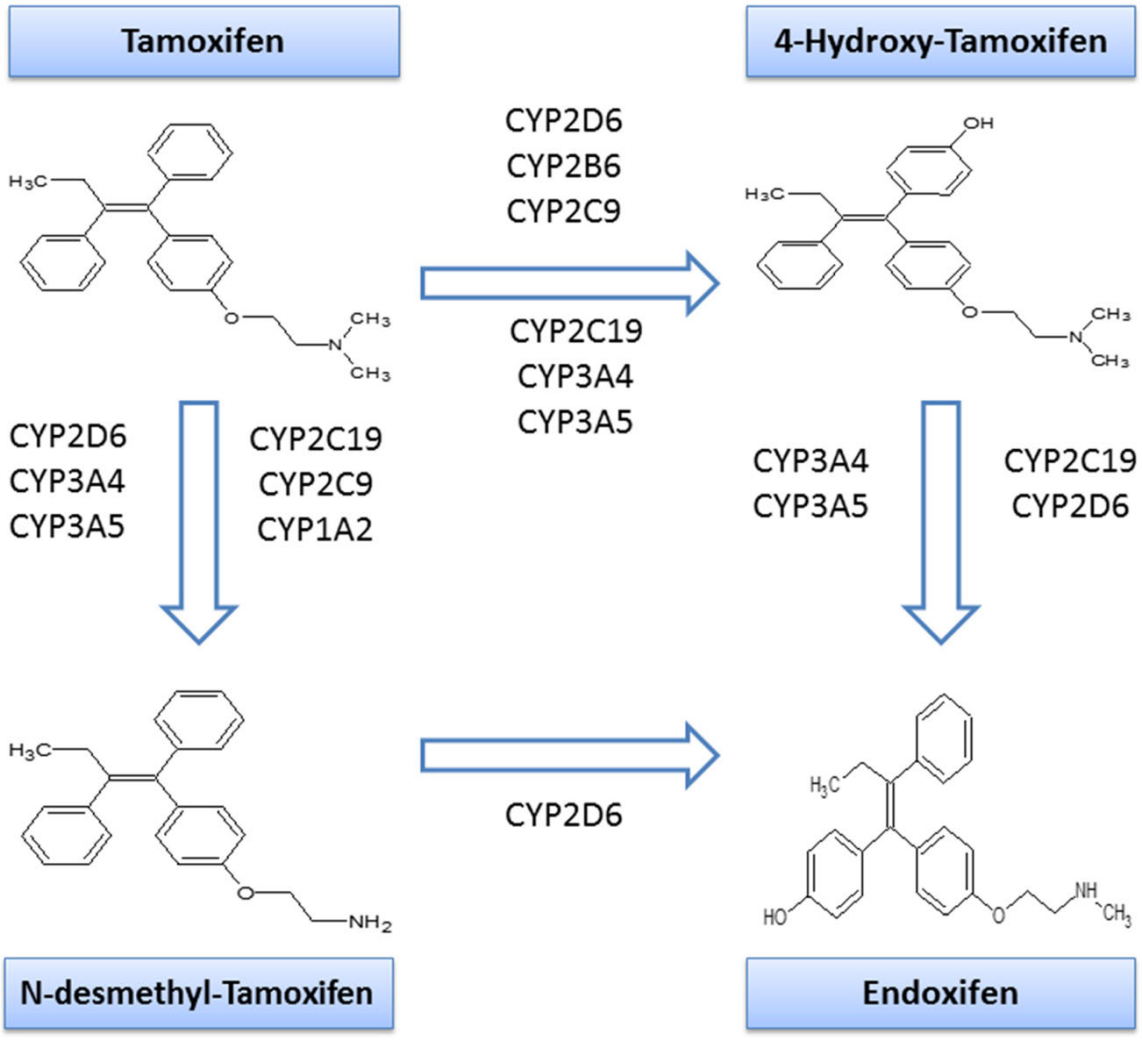
Tamoxifen metabolism

Tamoxifen has been widely prescribed to treat breast cancer patients with estrogen-receptor tumors for more than 40 years [3, 4]. As a prodrug, tamoxifen is metabolized by different cytochrome P-450 enzymes to its primary metabolites, 4-hydroxy-tamoxifen and *N*-desmethyl-tamoxifen [5] (NDM-tamoxifen). A second biotransformation from NDM-tamoxifen into endoxifen is principally regulated by CYP2D6 enzyme. At the same time, 4-hydroxy-tamoxifen also is biotransformed into endoxifen, mainly controlled by CYP3A4/5 and CYP2D6 enzymes, among others [6] (Fig. 1). Endoxifen is believed to be the most relevant tamoxifen metabolite since it is found in larger concentrations than 4-hydroxy-tamoxifen [7]. Additionally, CYP2D6 is considered the rate-limiting enzyme in tamoxifen metabolism [8] because it metabolizes the transformation of NDM-tamoxifen into endoxifen, which accounts for around 92% of tamoxifen metabolism [9]. However, it only partially explains the inter-patient variability of the metabolic ratio NDM-tamoxifen/endoxifen. According to Mürdter and colleagues [10], 68.7% of the variance in metabolic ratio of NDM-tamoxifen/endoxifen is explained by polymorphisms in CYP2D6.

Polymorphisms in genes encoding for other enzymes, such as CYP3A [11, 12], have been also related to tamoxifen metabolism. CYP3A4 is implicated in the metabolization of 30–50% of common therapeutic drugs [13], whereas CYP3A5 is also known to have a relevant function in drug metabolism [14].

CYP3A4 plays a role in the transformations of 4-hydroxy-tamoxifen to endoxifen, tamoxifen to NDM-tamoxifen, and tamoxifen to 4-hydroxy-tamoxifen (Fig. 1). Genetic polymorphisms of CYP3A4, with some effect on tamoxifen metabolism, have been identified [15, 16]. Still, there is limited information about the clinical relevance of most of these polymorphisms. However, CYP3A4*22 has been suggested to be an actionable CYP3A allele [17]. With a frequency of 5–7% in Caucasian population, CYP3A4*22 has been associated with decreased CYP3A4 activity [18]. CYP3A4*22 has been suggested to have a role in the metabolism of immunosuppressive drugs [18, 19], whereas for tamoxifen, diverse evidence can be found in the literature [20–22]. Teft et al. suggested that CYP3A4*22 carriers were two times more likely to have higher endoxifen levels [20]. Antunes et al. proposed that CYP3A4*22 genotype is associated with increased concentrations of 4-hydroxy-tamoxifen in the presence of impaired CYP2D6 activity [21]. In a clinical setting, Baxter and colleagues described CYP3A4*22 carriers tend to have less hot-flashes symptoms when compared with non-carriers [22].

CYP3A5 genetic polymorphisms are also involved in tamoxifen metabolism, but studies with tamoxifen have yielded conflicting data. Initially, Jin et al. described that carriers of non-functional CYP3A5 alleles, such as CYP3A5*3, were more likely to have higher endoxifen concentrations than individuals with a functional CYP3A5*1 allele [23]. Yet, no significant association between CYP3A5 polymorphisms with tamoxifen and its metabolites concentrations or clinical outcome has been found by other researchers [10, 20].

Combined data about the effect of CYP3A4 and CYP3A5 has also been analyzed in renal [19] and heart transplantation [24]. However, little is known about this combined effect on tamoxifen metabolism. In an attempt to elucidate the factors that are related to variability in tamoxifen metabolism, we aimed to investigate the effect of CYP3A4*22, CYP3A5*3, and *CYP3A* combined genotypes on tamoxifen metabolism.

## Methods

### Study population and study design

Blood and serum samples were used from individuals enrolled in the CYPTAM study (NTR 1509) [25]. The aim of the CYPTAM study was to correlate CYP2D6 predicted phenotypes and endoxifen with relapse-free survival, disease-free survival, and overall survival. In brief, from February 2008 till December 2010, patients with early breast cancer receiving adjuvant tamoxifen were recruited in the multicenter prospective CYPTAM study in The Netherlands and Belgium.

All the enrolled patients signed an informed consent. Women with a history of a previous malignancy within the last 5 years, with the exception of patients appropriately treated for an in situ cervix carcinoma or basal cell carcinoma, were excluded. Other exclusion criteria were pregnancy, breastfeeding, or an unwillingness to sign the informed consent. The CYPTAM study was approved by the Medical Ethical Committee of the Leiden University Medical Center in Leiden (The Netherlands). After inclusion in the CYPTAM study, and having used tamoxifen for more than 2 months but less than 1 year, both whole blood and serum samples were collected for genotyping and measurement of tamoxifen and metabolites concentrations, respectively. Trough levels were obtained 12 h after the last intake of tamoxifen.

### Metabolite measurements

Steady-state concentrations of tamoxifen and its metabolites (NDM-tamoxifen, 4-hydroxy-tamoxifen, and endoxifen) were measured in serum with high-performance liquid chromatography-tandem mass spectrometry (HPLC/MS/MS). This assay was developed and validated at the laboratory of Clinical Pharmacy and Toxicology at the Leiden University Medical Center and was described in detail earlier [26].

## Genotyping

### CYP2D6 genotyping

CYP2D6 genotyping was performed using the Amplichip CYP450 test (Roche Diagnostics, Indianapolis, USA) to test the major CYP2D6 alleles in DNA isolated from blood. All CYP2D6 genotypes were translated to predicted phenotypes according to Schroth and colleagues [27]. The considered CYP2D6 predicted phenotypes are as follows: ultra-rapid (UM, duplication of active alleles), extensive (EM, two fully functional alleles), heterozygous extensive (hetEM, one normal active allele with a non-functional allele), intermediate (IM, one non-functional allele with one decreased activity allele or two alleles with decreased activity), and poor metabolizers (PM, two non-functional alleles).

The CYP2D6 IM phenotype consisted of two alleles with decreased CYP2D6 activity and one non-functional allele combined with one allele with decreased CYP2D6 activity. Alleles with decreased CYP2D6 activity were *9, *10, *17, *29, *36, *41, *10xN, *17xN, and *41xN, whereas non-functional alleles were *3 until *8 alleles, *11, *14A, *15, *19, *20,*40, and *4xN″*.* As previously reported by Gaedigk et al. [28], the combination of a fully functional allele and a non-functional allele would most likely be translated as an EM phenotype. Still, this combination can also be considered as hetEM [27, 29], as previously described, and we used this term.

### CYP3A4/5 genotyping

CYP3A4*22 was analyzed with TaqMan 7500 (Applied Biosystems, Nieuwerkerk a.d. IJssel, The Netherlands) with predesigned assays, according to manufacturers’ protocol. CYP3A5*3 was determined with Pyrosequencer 96 MA (Isogen, IJsselstein, The Netherlands).

#### CYP3A combined genotypes

In order to investigate the combined effect of CYP3A4*22 and CYP3A5*3, genotype clusters were formed as follows:

1. Slow metabolizers (C1): metabolizers with at least one decreased activity allele in CYP3A4 (CYP3A4*22/*22 or CYP3A4*1/*22) and no CYP3A5 activity (CYP3A5*3/*3).
2. Intermediate metabolizers group 1 (C2): metabolizers with no decreased activity allele in CYP3A4 (CYP3A4*1/*1) and no CYP3A5 activity (CYP3A5*3/*3).
3. Intermediate metabolizers group 2 (C3): metabolizers with at least one decreased activity allele in CYP3A4 (CYP3A4*22/*22 or CYP3A4*1/*22) and at least one functional allele in CYP3A5 (CYP3A5*1/*1 or CYP3A5*1/*3).
4. Extensive metabolizers (C4): metabolizers with no decreased activity allele in CYP3A4 (CYP3A4*1/*1) and at least one functional allele in CYP3A5 (CYP3A5*1/*1 or CYP3A5*1/*3).

### Statistical analysis

Metabolic ratios were determined as concentration of substrate divided by concentration of metabolite. To analyze differences between metabolic ratios, a two-sided Student’s *t* test was used. To compare the concentrations of tamoxifen and its metabolites among CYP3A clusters, one-way ANOVA tests were used. For comparisons between tamoxifen and metabolite concentrations by CYP3A4 and CYP3A5 groups, a two-sided Student’s *t* test was performed. A multiple linear regression analysis was carried out to analyze the contributions of CYP3A4*22, CYP3A5*3, and the *CYP3A* combined genotypes to explain the total variability of the (log-transformed) metabolic ratios and concentrations of tamoxifen and its metabolites among treated patients. Statistical analyses were carried out with IBM SPSS for Windows, version 23.0. In analyses, test with *p* values <0.05 was considered to be statistically significant.

## Results

### Patient characteristics

A total of 667 female patients were enrolled in the CYPTAM study from February 2008 till December 2010 from 19 participating hospitals in The Netherlands and 6 hospitals in Belgium. The mean age of included patients was 56.4 years and in 79.5% were progesterone receptor-positive tumors. Table 1 lists the clinically and demographically relevant details of the CYPTAM patients.

**Table 1:** Baseline characteristics of the CYPTAM patients

		Total (*N* = 667 patients)
Age	Mean (years)	56.4
	Standard deviation (years)	11.1
Surgery	Mastectomy	310
	Breast conserving	352
	Not specified	5
Surgery axilla	Sentinel node procedure only	333
	Axillary lymph node dissection	329
	Not specified	5
Tumor stage	T1	356
	T2	274
	T3/T4	28
	Not specified	9
Nodal stage	N0	317
	N1	266
	N2	57
	N3	24
	Not specified	3
Histologic classification	Ductal adenocarcinoma	508
	Lobular adenocarcinoma	94
	Other	62
	Not specified	3
Histologic grade	G1	94
	G2	378
	G3	188
	Not specified	7
Progesterone receptor status	Positive	530
	Negative	127
	Not specified	10
HER2 receptor status	0	405
	1+	169
	2+	36
	3+	54
	Not specified	3
Adjuvant radiotherapy	Yes	462
	No	202
	Not specified	3
Adjuvant chemotherapy	Yes	407
	No	257
	Not specified	3

### CYP2D6 genotypes

Whole blood samples from 656 patients were available for genotyping. Of these, no genotype was obtained for 29 samples (4.4%), while for 637 patients (95.5%), CYP2D6 genotyping was successful, leading to a CYP2D6 predicted phenotype classification of 5 UMs (0.8%), 317 EMs (47.5%), 211 hetEMs (31.6%), 58 IMs (8.7%), and 47 PMs (7.0%).

### CYP3A4 genotypes

The cohort consisted of 563 (84.4%) CYP3A4*1/*1 carriers, 73 (10.9%) CYP3A4*1/*22 carriers, and 1 (0.1%) CYP3A4*22/*22 carrier. Unfortunately, genotyping failed in 30 samples (4.5%). CYP3A4 frequency and genotyping in the study population are shown in Table 2. Genotype distributions were in Hardy-Weinberg equilibrium and no linkage disequilibrium was observed between the CYP3A4*22 single nucleotide polymorphism (SNP) and the CYP3A5*3 allele (LD < 0.1).

**Table 2:** Genotype distribution and frequency in the study population

Genotypes		Total individuals (*n*)	Frequency (%)
*CYP3A4*	*1/*1	563	84.4
	*1/*22	73	10.9
	*22/*22	1	0.1
	Unknown	30	4.5
*CYP3A5*	*3/*3	554	83.1
	*1/*3	94	14.1
	*1/*1	4	0.6
	Unknown	15	2.2
CYP3A4/CYP3A5 cluster	C1	63	9.4
	C2	471	70.6
	C3	10	1.5
	C4	88	13.2
	Unknown	35	5.2

C1, CYP3A4*22 carriers and CYP3A5*1 non-carriers; C2, CYP3A4*22 non-carriers and CYP3A5*1 non-carriers; C3, CYP3A4*22 carriers and CYP3A5*1 carriers; C4, CYP3A4*22 non-carriers and CYP3A5*1 carriers; Unknown, not genotyped or missing data

### CYP3A5 genotypes

Frequencies and distribution in the study population are listed in Table 2. The most frequent genotype was CYP3A5*3/*3, followed by CYP3A5*1/*3 and CYP3A5*1/*1, consisting of 554 (83.1%), 94 (14.1%), and 4 patients (0.6%), respectively. In 15 cases (2.2%), no genotype was obtained. Genotype distributions were in Hardy-Weinberg equilibrium and no linkage disequilibrium was observed between the CYP3A4*22 SNP and the CYP3A5*3 allele (LD < 0.1).

### CYP3A4/CYP3A5 genotype clusters

C1, C2, C3, and C4 clusters were formed as described to analyze the additional combined effect of the CYP3A4 and CYP3A5 genotype on the CYP2D6 genotype. C1 consisted of 63 individuals (9.4%), 471 individuals for C2 (70.6%), 10 cases for C3 (1.5%), and 88 cases for C4 (13.2%). In 35 cases, no combined cluster could be made due to previous missing data.

### Association of tamoxifen and its metabolites to CYP3A4 genotype, CYP3A5 genotype, and CYP3A4/5 combined genotypes

A substantial variation in the metabolic ratios of tamoxifen and its metabolites between individuals was observed. An overview of the mean and standard deviations (SD) of tamoxifen and its metabolite metabolic ratios by CYP3A4, CYP3A5 genotypes and CYP3A clusters is presented in Table 3.

**Table 3:** Summary of CYP3A4 and CYP3A5 covariate analysis

		*R* ^2^	*p* value
Ln (MR tamoxifen/NDM-tamoxifen)	*CYP2D6*	0.218	<0.001
	*CYP2D6* and CYP3A4*22	0.239	<0.001
	*CYP2D6* and CYP3A5*3	0.221	0.35
	*CYP2D6* and *CYP3A* cluster	0.224	0.013
Ln MR tamoxifen/4-hydroxy-tamoxifen	*CYP2D6*	0.219	<0.001
	*CYP2D6* and CYP3A4*22	0.214	0.715
	*CYP2D6* and CYP3A5*3	0.223	0.947
	*CYP2D6* and *CYP3A* cluster	0.217	0.908
Ln MR 4-hydroxy-tamoxifen/endoxifen	*CYP2D6*	0.449	<0.001
	*CYP2D6* and CYP3A4*22	0.456	0.116
	*CYP2D6* and CYP3A5*3	0.462	0.038
	*CYP2D6* and *CYP3A* cluster	0.465	0.016
Ln MR NDM-tamoxifen/endoxifen	*CYP2D6*	0.570	<0.001
	*CYP2D6* and CYP3A4*22	0.574	0.375
	*CYP2D6* and CYP3A5*3	0.581	0.477
	*CYP2D6* and *CYP3A* cluster	0.579	0.779

MR = metabolic ratio. Ln(MR tamoxifen/NDM-tamoxifen) = natural log of MR tamoxifen/NDM-tamoxifen; Ln(MR tamoxifen/4-hydroxy-tamoxifen) = natural log of MR tamoxifen/4-hydroxy-tamoxifen; Ln(MR 4-hydroxy-tamoxifen/endoxifen) = natural log of MR 4-hydroxy-tamoxifen/endoxifen; Ln(MR NDM-tamoxifen/endoxifen) = natural log of MR NDM-tamoxifen/endoxifen

**Fig. 2:**
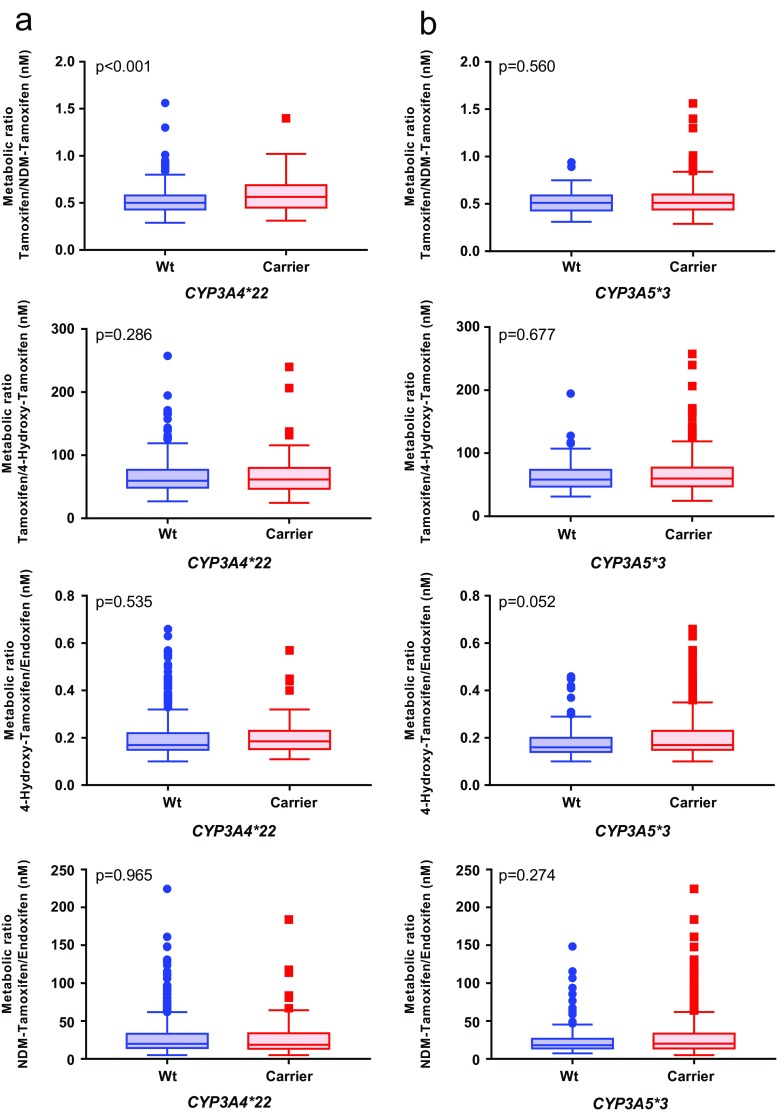
Association of CYP3A4 and CYP3A5 genotypes with tamoxifen and its metabolite metabolic ratios. (a) Association between CYP3A4*22/*22 and CYP3A4*22/*1 or CYP3A4*1/*1 carriers with tamoxifen and its metabolite metabolic ratios. (b) Association between CYP3A5*3/*3 and CYP3A5*3/*1 or CYP3A5*1/*1 carriers with tamoxifen and its metabolite metabolic ratios

The metabolic ratio tamoxifen/NDM-tamoxifen was statistically different (*p* < 0.001) between CYP3A4*22/*22 and CYP3A4*1/*22 or CYP3A4*1/*1 individuals, whereas other metabolic ratios (tamoxifen/4-hydroxy-tamoxifen, 4-hydroxy-tamoxifen/endoxifen, and NDM-tamoxifen) did not show any difference. The metabolic ratios of tamoxifen did not show any difference between CYP3A5*1/*3 or CYP3A5*1/*1 and CYP3A5*3/*3 individuals (*p* > 0.05). Figure 2 shows the comparisons of tamoxifen and its metabolite metabolic ratios stratified by the CYP3A4 and CYP3A5 genotypes.

**Fig. 3:**
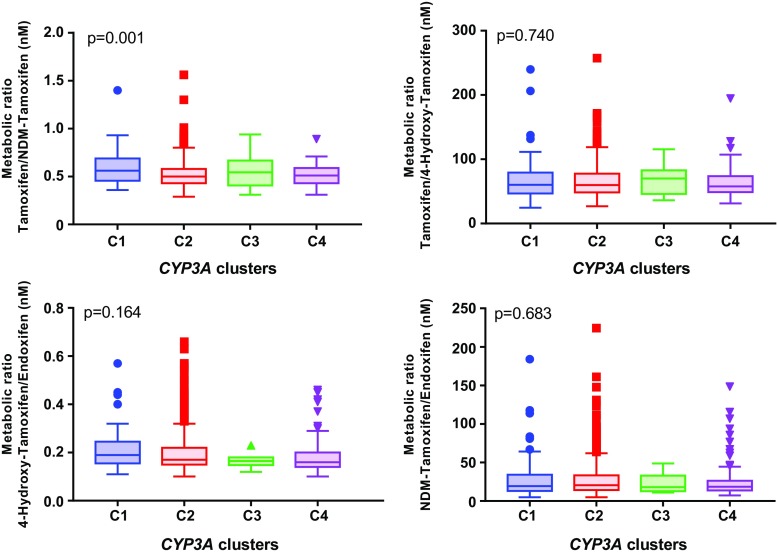
Association of CYP3A4 and CYP3A5 genotypes with tamoxifen and its metabolite metabolic ratios. C1, CYP3A4*22 carriers and CYP3A5*1 non-carriers; C2, CYP3A4*22 non-carriers and CYP3A5*1 non-carriers; C3, CYP3A4*22 carriers and CYP3A5*1 carriers; C4, CYP3A4*22 non-carriers and CYP3A5*1 carriers

At the same time, only the metabolic ratio of tamoxifen/NDM-tamoxifen was significantly different among CYP3A4/5 combined genotypes (C1, C2, C3, and C4) (*p* < 0.001). The other metabolic ratios (tamoxifen/4-hydroxy-tamoxifen, 4-hydroxy-tamoxifen/endoxifen, and NDM-tamoxifen/endoxifen) did not significantly differ between the different CYP3A4/5 clusters. Figure 3 presents a comparison between the different CYP3A4/5 clusters by the diverse metabolic ratios.

The mean concentrations of tamoxifen, 4-hydroxy-tamoxifen, and NDM-tamoxifen of CYP3A4*22 carriers were statistically higher (*p* < 0.05). Endoxifen mean concentrations were not statistically higher (*p* = 0.088), but a trend toward higher endoxifen concentrations was observed among CYP3A4*22 individuals. An overview of mean concentrations of tamoxifen and its metabolites in the different groups is presented in Supplementary Table 1 and Supplementary Figs 1 and 2.

### Association between metabolic ratios of tamoxifen and its metabolites to *CYP2D6, CYP3A4/5*, and combined genotypes

The explained variability (*R* ^2^) of (log-transformed) metabolic ratios of tamoxifen/NDM-tamoxifen, tamoxifen/4-hydroxy-tamoxifen, 4-hydroxy-tamoxifen/endoxifen, and NDM-tamoxifen/endoxifen due to genetic variations in *CYP2D6* was 21.8%, 21.9%, 44.9%, and 57.0%, respectively.

A multiple linear regression indicated a combined analyses accounting for *CYP2D6* and *CYP3A4* (CYP3A4*22 and CYP3A4*1) genotypes significantly improved the prediction of the metabolic ratio tamoxifen/NDM-tamoxifen from 21.8 to 23.9%, whereas the explained variability for other metabolic ratios only showed marginal improvements.

Another multiple linear regression was used to test the effect of *CYP2D6* and *CYP3A5* (CYP3A5*3 and CYP3A5*1) genotypes together. However, no statistically significant difference of the explained variability was found (*p* > 0.05) compared to *CYP2D6* alone.

In a third linear regression, the combined role of *CYP2D6* and *CYP3A* clusters (C1, C2, C3, and C4) together was tested. Still, no significant improvements in the explained variability (*R* ^2^) were observed. A summary of *CYP3A4*, *CYP3A5*, and *CYP3A* covariate analysis is presented in Table 4.

**Table 4:** Overview of the means and standard deviations of tamoxifen, and its metabolite concentrations and metabolic ratios according to *CYP3A4*, *CYP3A5* genotypes and *CYP3A* cluster

Tamoxifen and its metabolite metabolic ratios according to *CYP3A4*, *CYP3A5* genotypes and *CYP3A* cluster				
	MR tamoxifen/NDM-tamoxifenMean (SD)	MR0 tamoxifen/4-hydroxy-tamoxifenMean (SD)	MR 4-hydroxy-tamoxifen/endoxifenMean (SD)	MR NDM-tamoxifen/endoxifenMean (SD)
CYP3A4 genotypes (*n* = 632)				
CYP3A4*22/*22 and CYP3A4*1/*22 (*n* = 560)	0.59 (0.19)	68.6 (35.00)	0.21 (0.08)	29.3 (29.50)
CYP3A4*1/*1 (*n* = 72)	0.52 (0.13)	65.0 (25.40)	0.20 (0.09)	29.1 (25.50)
*p* value	<0.001	0.286	0.535	0.965
CYP3A5 genotypes (*n* = 647)				
CYP3A5*1/*3 or CYP3A5*1/*1 (*n* = 97)	0.52 (0.12)	64.16 (24.53)	0.19 (0.08)	26.49 (24.10)
CYP3A5*3/*3 (*n* = 550)	0.53 (0.14)	65.38 (26.93)	0.21 (0.09)	29.64 (26.55)
*p* value	0.560	0.677	0.052	0.274
CYP3A cluster genotypes (*n* = 626)				
Slow (C1; *n* = 61)	0.59 (0.18)	68.45 (36.66)	0.21 (0.08)	30.57 (31.46)
IM1 (C2; *n* = 469)	0.52 (0.13)	65.36 (25.55)	0.20 (0.09)	29.48 (25.57)
IM2 (C3; *n* = 10)	0.56 (0.19)	68.20 (25.85)	0.17 (0.03)	22.82 (12.91)
Extensive (C4; *n* = 87)	0.52 (0.11)	63.70 (24.48)	0.19 (0.08)	26.91 (25.09)
*p* value	<0.001	0.740	0.164	0.683

MR, metabolic ratio; SD, standard deviation; slow group (C1), CYP3A4*22 carriers and CYP3A5*1 non-carriers; intermediate 1 group (C2), CYP3A4*22 non-carriers and CYP3A5*1 non-carriers; intermediate 2 group (C3), CYP3A4*22 carriers and CYP3A5*1 carriers; extensive group (C4), CYP3A4*22 non-carriers and CYP3A5*1 carriers

The explained variability (*R* ^2^) of (log-transformed) concentrations of tamoxifen, endoxifen, 4-hydroxy-tamoxifen, and NDM-tamoxifen due to genetic variations in *CYP2D6*, *CYP3A4*, and *CYP3A5* genotype, and *CYP3A* combined genotypes is presented in Supplementary Table 2. The explained variability of (log-transformed) concentrations of endoxifen due to CYP3A4*22 genotype marginally increased from 42.3 to 42.8% (*p* < 0.001).

## Discussion

In the present study, the contribution of CYP3A4*22, CYP3A5*3, and combined genotypes to the metabolism of tamoxifen and the formation of the active metabolite endoxifen was investigated. Our data show that CYP3A4*22 genotype slightly contributes to explaining the pharmacokinetic variability between patients receiving tamoxifen, but the effect is small. CYP3A5*3 genotype and *CYP3A4/5* combined genotypes do not significantly help to improve the explained variability in tamoxifen metabolism.

The explained variability (*R* ^2^) of (log-transformed) endoxifen concentrations due to CYP2D6 predicted phenotypes was 42.3%, while 57.0% of the variability in metabolic ratio NDM-tamoxifen/endoxifen was explained by CYP2D6. Previously, Mürdter and colleagues reported a high, 68.7% variance in metabolic ratio of NDM-tamoxifen/endoxifen due to genetic variations in *CYP2D6* genotype [10]. In our study, we observed a lower variance of metabolic ratio of NDM-tamoxifen/endoxifen (57.0%); however, the data demonstrate that CYP2D6 genotype alone only partially explains the variability between patients using tamoxifen.

In this study, when the CYP3A4*22 genotype was taken into account, in addition to the CYP2D6 genotype, the explained variability (*R* ^2^) of (log-transformed) endoxifen concentrations slightly improved from 42.3 to 42.8% (*p* < 0.001), whereas the explained variability of the metabolic ratio NDM-tamoxifen/endoxifen did not significantly increase (from 57.0 to 57.4%, *p* = 0.375). Interestingly, the explained variability of (log-transformed) metabolic ratio tamoxifen/NDM-tamoxifen was found to be slightly increased if the CYP3A4*22 genotype was added to the analysis (improvement from 21.8 to 23.9%, *p* < 0.001). A higher metabolic ratio tamoxifen/NDM-tamoxifen was also noted in CYP3A4*22 carriers (0.59 vs. 0.52, *p* < 0.001). At the same time, our data showed that CYP3A4*22 carriers have a statistically significant higher mean concentration of tamoxifen, 4-hydroxy-tamoxifen, and NDM-tamoxifen (*p* < 0.05), while a trend toward higher endoxifen concentrations was observed (*p* = 0.088).

Our results are in line with the previous conclusions by Teft et al. [20] and Antunes and colleagues [21]. In both studies, higher mean concentrations of tamoxifen and its metabolites were unexpectedly measured in CYP3A4*22 carriers. At first glance, a decreased CYP3A4 activity may lead to a diminished transformation of tamoxifen into its active metabolites, and consequently, lower concentrations could be expected. On the contrary, higher concentrations of tamoxifen and its metabolites were found.

A potential explanation for these findings could be due to decreased CYP3A4 activity and a larger intestinal and hepatic bioavailability of tamoxifen in the CYP3A4*22 individuals [20]. According to Teft and colleagues [20], CYP3A4*22 carriers would have a reduced intestinal CYP3A4 activity and higher tamoxifen bioavailability, which would result in higher levels of unmetabolized tamoxifen. At the same time, a diminished CYP3A4 action at hepatic level would mean a diminished hepatic first-pass metabolism of tamoxifen, which would be translated in higher remaining concentrations of tamoxifen available for further transformations into 4-hydroxy-tamoxifen and NDM-tamoxifen. Moreover, Antunes et al. suggested that the reduced tamoxifen metabolism resulting from CYP3A4*22 is probably compensated by other enzymes, whereas the transformation from tamoxifen into 4-hydroxy-tamoxifen would be more relevant in CYP3A4*22 carriers when CYP2D6 activity is decreased [21]. Although this hypothesis appears plausible, we did not observe any significant difference in metabolic ratios tamoxifen/4-hydroxy-tamoxifen and 4-hydroxy-tamoxifen/endoxifen between CYP3A4*22 and CYP3A4*1 carriers after adjustment for CYP2D6 activity.

In the present study, the CYP3A5*3 genotype does not significantly contribute to explaining the inter-variability among patients treated with tamoxifen. Only CYP3A5*3 marginally improved the explained variance of the (log-transformed) metabolic ratio 4-hydroxy-tamoxifen/endoxifen (from 44.9 to 46.2%, *p* < 0.038). However, we did not find any statistically significant differences in mean concentrations of tamoxifen and its metabolites, nor in the mean metabolic ratios between CYP3A5*3 and CYP3A5*1 individuals. Jin and colleagues found that CYP3A5*3 carriers treated with tamoxifen reached higher endoxifen concentrations than CYP3A5*1 individuals [23]. Our results, however, are in line with the results of Tucker et al., who did not see significant variations in tamoxifen and its metabolite concentrations among CYP3A5*3 and CYP3A5*1 carriers [30]. In a clinical context, several conflicting results have been published, showing disparate findings. According to Wegman and colleagues, CYP3A5*3 homozygous carriers tend to have an increased risk of recurrence, albeit not statistically significant [31].

In the same way, our findings suggested that CYP3A combined genotypes do not significantly contribute to explaining the variability between individuals treated with tamoxifen, with the exception of the (log-transformed) metabolic ratio tamoxifen/NDM-tamoxifen (*p* < 0.001). The slow metabolizer C1 group, consisting of CYP3A4*22 carriers and the non-functional CYP3A5*3 allele, showed higher metabolic ratios of tamoxifen/NDM-tamoxifen compared to the other groups (C2, C3, and C4). These results might be clarified by the previously described difference in metabolic ratio in the CYP3A4*22 individuals and therefore in the CYP3A combined genotypes.

A potential limitation of our analysis might be due to the use of CYP3A4/5 inhibitors during the study, as CYP3A4/5 activity can be influenced. Unfortunately, information about concomitant medicines was not systematically evaluated and consequently available data were too sparse for analysis.

In conclusion, our data demonstrated that CYP3A genotype slightly contributes to explaining the variability between patients in tamoxifen metabolism; however, the effect is small, and therefore, it is unlikely to have any significant clinical relevance for the efficacy of tamoxifen.

## Electronic supplementary material

Supplementary Fig. 1 (DOCX 45 kb)

Supplementary Fig. 2 (DOCX 27 kb)

Supplementary Table 1 (DOCX 15 kb)

Supplementary Table 2 (DOCX 15 kb)
