# Supplementary material for: Effect of CYP3A4*22, CYP3A5*3, and CYP3A combined genotypes on tamoxifen metabolism
Source: Eur J Clin Pharmacol. 2017 Aug 28;73(12):1589–98. doi: 10.1007/s00228-017-2323-2 (PMC5684327; doi:10.1007/s00228-017-2323-2)
Supplement: Supplementary file 1 — (DOCX 45 kb) [file 228_2017_2323_MOESM1_ESM.docx]

**Figure 1. Influence of CYP3A4 and CYP3A5 genotype on tamoxifen and its metabolites concentrations.**

1. Association between *CYP3A4*22/*22* and *CYP3A4*22/*1* or *CYP3A4*1/*1* carriers with through concentrations of tamoxifen, 4-hydroxy-tamoxifen, NDM-tamoxifen and endoxifen.
2. Association between *CYP3A5*3/*3* and *CYP3A5*3/*1* or *CYP3A5*1/*1* carriers with through concentrations of tamoxifen, 4-hydroxy-tamoxifen, NDM-tamoxifen and endoxifen.
