# Supplementary material for: Effect of CYP3A4*22, CYP3A5*3, and CYP3A combined genotypes on tamoxifen metabolism
Source: Eur J Clin Pharmacol. 2017 Aug 28;73(12):1589–98. doi: 10.1007/s00228-017-2323-2 (PMC5684327; doi:10.1007/s00228-017-2323-2)
Supplement: Supplementary file 2 — (DOCX 27 kb) [file 228_2017_2323_MOESM2_ESM.docx]

**Figure 2. Influence of CYP3A4/5 combined genotype on tamoxifen and its metabolites concentrations.**

Concentrations levels of tamoxifen and its metabolites comparing CYP3A4/5 combined genotypes: C1, *CYP3A4*22* carriers and *CYP3A5*1* non-carriers; C2, *CYP3A4*22* non-carriers and *CYP3A5*1* non-carriers; C3, *CYP3A4*22* carriers and *CYP3A5*1* carriers; C4, *CYP3A4*22* non-carriers and *CYP3A5*1* carriers
