# Supplementary material for: Effect of CYP3A4*22, CYP3A5*3, and CYP3A combined genotypes on tamoxifen metabolism
Source: Eur J Clin Pharmacol. 2017 Aug 28;73(12):1589–98. doi: 10.1007/s00228-017-2323-2 (PMC5684327; doi:10.1007/s00228-017-2323-2)
Supplement: Supplementary file 3 — (DOCX 15 kb) [file 228_2017_2323_MOESM3_ESM.docx]

**Table 1. Overview of the means and standard deviations of tamoxifen, and its metabolites concentrations according to *CYP3A4, CYP3A5* genotypes and *CYP3A* cluster**. SD=Standard deviation; Slow group (C1), *CYP3A4*22* carriers and *CYP3A5*1* non-carriers; Intermediate 1 group (C2), *CYP3A4*22* non-carriers and *CYP3A5*1* non-carriers; Intermediate 2 group (C3), *CYP3A4*22* carriers and *CYP3A5*1* carriers; Extensive group (C4), *CYP3A4*22* non-carriers and *CYP3A5*1* carriers.

| **CYP3A4 genotypes (n=632)** | **Tamoxifen mean concentration (nM) (SD)** | **Endoxifen mean concentration (nM) (SD)** | **4-Hydroxy-Tamoxifen**  **Mean concentration (nM) (SD)** | **NDM-Tamoxifen**  **Mean concentration (nM) (SD)** |
| --- | --- | --- | --- | --- |
| ***CYP3A4*22/*22 and CYP3A4*1/*22* (n=560)** | 370.64 (115.92) | 32.19 (17.94) | 5.95 (2.75) | 650.83 (280.34) |
| ***CYP3A4*1/*1* (n=72)** | 303.81 (115.92) | 28.83 (15.38) | 5.05 (2.14) | 594.75 (213.87) |
| **p-value** | p<0.001 | p=0.088 | p<0.001 | p=0.044 |
|  |  |  |  |  |
| **CYP3A5 genotypes (n=647)** | **Tamoxifen concentration (nM) (SD)** | **Endoxifen concentration (nM) (SD)** | **4-Hydroxy-Tamoxifen concentration (nM) (SD)** | **NDM-Tamoxifen concentration (nM) (SD)** |
| ***CYP3A5*1/*3 or CYP3A5*1/*1*(n=97)** | 301.13 (131.38) | 30.50 (16.2) | 5.04 (2.27) | 585.81 (219.98) |
| ***CYP3A5*3/*3*(n=550)** | 312.70 (119.79) | 29.09 (15.67) | 5.19 (2.23) | 603.35 (223.72) |
| **p-value** | p=0.388 | p=0.418 | p=0.528 | p=0.476 |
|  |  |  |  |  |
| **CYP3A cluster genotypes (n=626)** | **Tamoxifen concentration (nM) (SD)** | **Endoxifen concentration (nM) (SD)** | **4-Hydroxy-Tamoxifen (nM)**  **(SD)** | **NDM-Tamoxifen concentration (nM) (SD)** |
| **Slow (C1; n=61)** | 379.41 (146.11) | 32.71 (18.53) | 6.14 (2.79) | 671.50 (287.03) |
| **IM1 (C2; n=469)** | 304.57 (113.45) | 28.51 (15.14) | 5.04 (2.09) | 595.27 (213.33) |
| **IM2 (C3; n=10)** | 305.74 (148.30) | 29.15 (15.42) | 4.82 (2.52) | 542.34 (226.56) |
| **EXTENSIVE (C4; n=87)** | 300.6 (130.24) | 30.65 (16.38) | 5.06 (2.25) | 590.80 (220.00) |
| **p-value** | p<0.001 | p=0.194 | p=0.003 | p=0.064 |
