# Supplementary material for: Effect of CYP3A4*22, CYP3A5*3, and CYP3A combined genotypes on tamoxifen metabolism
Source: Eur J Clin Pharmacol. 2017 Aug 28;73(12):1589–98. doi: 10.1007/s00228-017-2323-2 (PMC5684327; doi:10.1007/s00228-017-2323-2)
Supplement: Supplementary file 4 — (DOCX 15 kb) [file 228_2017_2323_MOESM4_ESM.docx]

**Table 2. Summary of *CYP3A4/5* genotype and *CYP3A* combined genotypes covariate analysis**

Ln(Tamoxifen)= natural log of tamoxifen concentration; Ln(Endoxifen)= natural log of endoxifen concentration; Ln(4-Hydroxy-Tamoxifen)= natural log of 4-hydroxy-tamoxifen concentration; Ln(NDM-Tamoxifen)= natural log of NDM-tamoxifen concentration.

|  |  | **R^2^** | **p-value** |
| --- | --- | --- | --- |
| **Ln Tamoxifen** | ***CYP2D6*** | 0.003 | 0.169 |
|  | ***CYP2D6* and *CYP3A4*22*** | 0.024 | <0.001 |
|  | ***CYP2D6* and *CYP3A5*3*** | 0.007 | 0.182 |
|  | ***CYP2D6* and *CYP3A* cluster** | 0.015 | 0.010 |
| **Ln Endoxifen** | ***CYP2D6*** | 0.423 | <0.001 |
|  | ***CYP2D6* and *CYP3A4*22*** | 0.428 | <0.001 |
|  | ***CYP2D6* and *CYP3A5*3*** | 0.425 | 0.933 |
|  | ***CYP2D6* and *CYP3A* cluster** | 0.427 | 0.439 |
| **Ln 4-Hydroy-Tamoxifen** | ***CYP2D6*** | 0.127 | <0.001 |
|  | ***CYP2D6* and *CYP3A4*22*** | 0.140 | <0.001 |
|  | ***CYP2D6* and *CYP3A5*3*** | 0.127 | 0.174 |
|  | ***CYP2D6* and *CYP3A* cluster** | 0.132 | 0.013 |
| **Ln NDM-Tamoxifen** | ***CYP2D6*** | 0.138 | <0.001 |
|  | ***CYP2D6* and *CYP3A4*22*** | 0.141 | 0.181 |
|  | ***CYP2D6* and *CYP3A5*3*** | 0.146 | 0.359 |
|  | ***CYP2D6* and *CYP3A* cluster** | 0.145 | 0.162 |
